# Mutational landscape of phenylketonuria in Iran

**DOI:** 10.1111/jcmm.17865

**Published:** 2023-07-31

**Authors:** Naser Ajami, Anvar Soleimani, Reza Jafarzadeh‐Esfehani, Mojtaba Hasanpour, Romina Rashid Shomali, Mohammad Reza Abbaszadegan

**Affiliations:** ^1^ Department of Medical Genetics and Molecular Medicine, School of Medicine Mashhad University of Medical Sciences Mashhad Iran; ^2^ Medical Genetics Research Center, School of Medicine Mashhad University of Medical Sciences Mashhad Iran; ^3^ Department of Medical Microbiology, College of Health Sciences Cihan University‐ Sulaimaniya Sulaimaniya Iraq; ^4^ Blood Borne Infections Research Center, Academic Center for Education Culture and Research (ACECR)‐ Khorasan Razavi Mashhad Iran; ^5^ Department of Biology, Faculty of Sciences University of Guilan Rasht Iran; ^6^ Reproductive Health Research Center, Department of Obstetrics and Gynecology, Alzahra Hospital Guilan University of Medical Sciences Rasht Iran; ^7^ Immunology Research Center Mashhad University of Medical Sciences Mashhad Iran

**Keywords:** Iranian populations, mutation profile, phenylalanine hydroxylase, phenylketonuria

## Abstract

To date more than 1000 different variants in the *PAH* gene have been identified in patients with phenylketonuria (PKU). In Iran, several studies have been performed to investigate the genetics bases of the PKU in different parts of the country. In this study, we have analysed and present an update of the mutational landscape of the *PAH* gene as well as the population genetics and frequencies of detected variants for each cohort. Published articles on PKU mutations in Iran were identified through a comprehensive PubMed, Google Scholar, Web of Science (ISI), SCOPUS, Elsevier, Wiley Online Library and SID literature search using the terms: “phenylketonuria”, “hyperphenylalaninemia”, and “PKU” in combination with “Iran”, “Iranian population”, “mutation analysis”, and “Molecular genetics”. Among the literature‐related to genetics of PKU, 18 studies were on the PKU mutations. According to these studies, in different populations of Iran 1497 patients were included for mutation detection that resulted in detection of 129 different mutations. Results of genetic analysis of the different cohorts of Iranian PKU patients show that the most prevalent mutation in Iran is the pathogenic splice variant c.1066‐11G > A, occurring in 19.54% of alleles in the cohort. Four other common mutations were p.Arg261Gln, p.Pro281Leu, c.168 + 5G > C and p.Arg243Ter (8.18%, 6.45%, 5.88% and 3.7%, respectively). One notable feature of the studied populations is its high rate of consanguineous marriages. Considering this feature, determining the prevalent PKU mutations could be advantageous for designing screening and diagnostic panels in Iran.

## 1 INTRODUCTION

Phenylketonuria (PKU; OMIM #261600) is an autosomal recessive inborn error of phenylalanine metabolism caused by wide range of mutations of the phenylalanine hydroxylase (*PAH*) gene located on chromosome 12q22–24.1 coding for the hepatic enzyme PAH that converts phenylalanine into tyrosine in the presence of molecular oxygen and catalytic amounts of tetrahydrobiopterin (BH4).^1^ PKU as epitome of human biochemical genetics^2^ can historically claim some ‘firsts’ in this era: the first in which a toxic agent, high phenylalanine concentration in blood, found to cause intellectual disability^3^; the first for which the biochemical background was understood; the first for which successful treatment was made available^4^; the first to be controlled by dietary management; the first to be detected by newborn screening^5^; and also PKU is a primary application for human gene therapy. PKU is therefore considered as a paradigm for monogenic metabolic disorders.^6^

In 98% of PKU patients, defects of the PAH enzyme are due to mutations in the *PAH* gene. about 1%–2% of cases, mutations occur in the genes that encode enzymes for biosynthesis or regeneration of BH4, an obligatory cofactor required for the activity of PAH.^1^ The human *PAH* gene spans 90 kb (about 171 kb with flanking regions) and contains 13 exons which encodes for a polypeptide of 452 amino acids.^7^ Over some four decades of research, several studies have listed the *PAH* gene variants occurring in all exons, but most commonly in exons 3, 6, 7 and 11.^8^ To date more than 1723 variants in the *PAH* gene have been recorded in BIOPKU (http://www.biopku.org) database.^9^ The huge number of *PAH* mutations and the wide variable distribution of frequent mutations between geographical regions and ethnic groups makes PKU a genetic disease with striking degree of allelic heterogeneity.^10^

Clinical manifestations of PKU are related to the toxic accumulation of phenylalanine in the blood and brain and based on the degree of PAH deficiency in a spectrum of disorders including classic PKU, mild PKU and mild hyperphenylalaninaemia (HPA). Classic PKU is a result of complete or near‐complete deficiency of PAH activity and if the disease left untreated and/or late‐diagnosed, the neurotoxic effects of elevated phenylalanine and its by‐products can lead to irreversible severe neurodevelopmental delay and progressive intellectual disability accompanied by several additional symptoms, which may include eczematous rashes, seizures, autism, hyperactivity and motor deficits at an early age. Developmental problems as well as psychiatric symptoms often become apparent as the child grows. Mild PKU and mild HPA are associated with lower risk of neurological, and cognitive abnormalities in the absence of treatment.^11^

PKU diagnosis is achieved soon after birth through newborn screening programs using the Guthrie card blood spot obtained from a heel prick. Early identification and treatment prevent the neurotoxic effects of elevated phenylalanine and its metabolites.^12^ Changing PKU from a disease of severe intellectual disability to a disease with almost normal development, phenylalanine restricted dietary is the main strategy for the standard treatment of the disease.^13^ The novel therapeutic approaches including BH4, large neutral amino acids, phenylalanine ammonia lyase, chaperone treatment may in part replace dietary restriction.^13^ Among these therapeutic modalities, supplementation of BH4, is the only alternative therapy that currently have been approved. This treatment is beneficial only in a proportion (at least 20%–30%) of PKU patients (so‐called BH4‐responsive) especially in milder PKU phenotypes.^14^ One of the potential modifiers of BH4 treatment effectiveness is *PAH* genotype and although this modifier cannot predict BH4 responsiveness with 100% accuracy, specific mutations resulting residual PAH activity is strongly associated with the BH4‐responsive phenotype.^15^

PKU is one of the most common inherited disorders in Iran with an incidence of roughly 1:4698.^16^ As PKU has autosomal recessive inheritance, consanguineous marriage is an important risk factor; thus, high disease incidence is expectable for countries with a high rate of consanguineous marriages.^16^ The genetic basis of PKU in the Iranian population has been investigated in studies done separately among different Iranian ethnic groups. Herein, we have tried to combine data from these studies and present an update of the mutational landscape of the PKU in Iran.

## 2 DESIGN OF THE STUDY

Published articles on PKU mutations in Iran were identified through a comprehensive electronic search using the databases MEDLINE (via PubMed), Google Scholar, Web of Science (ISI), SCOPUS, Elsevier, Wiley Online Library and SID literature search using the terms: “phenylketonuria”, “hyperphenylalaninemia”, and “PKU” in combination with “Iran”, “Iranian population”, “mutation analysis”, and “Molecular genetics”. We gave preference to papers published during 2003–2023 covering the genetics of PKU in different regions of the country. Although the Persian language publications were also accepted, but were replaced by two newly searched English publications with the same number of patients and the same authors, which were more complete than their Persian publications. All original articles that directly reported mutations of PKU in the Iranian populations were included.

## 3 RESULTS AND DISCUSSION

**FIGURE 1:**
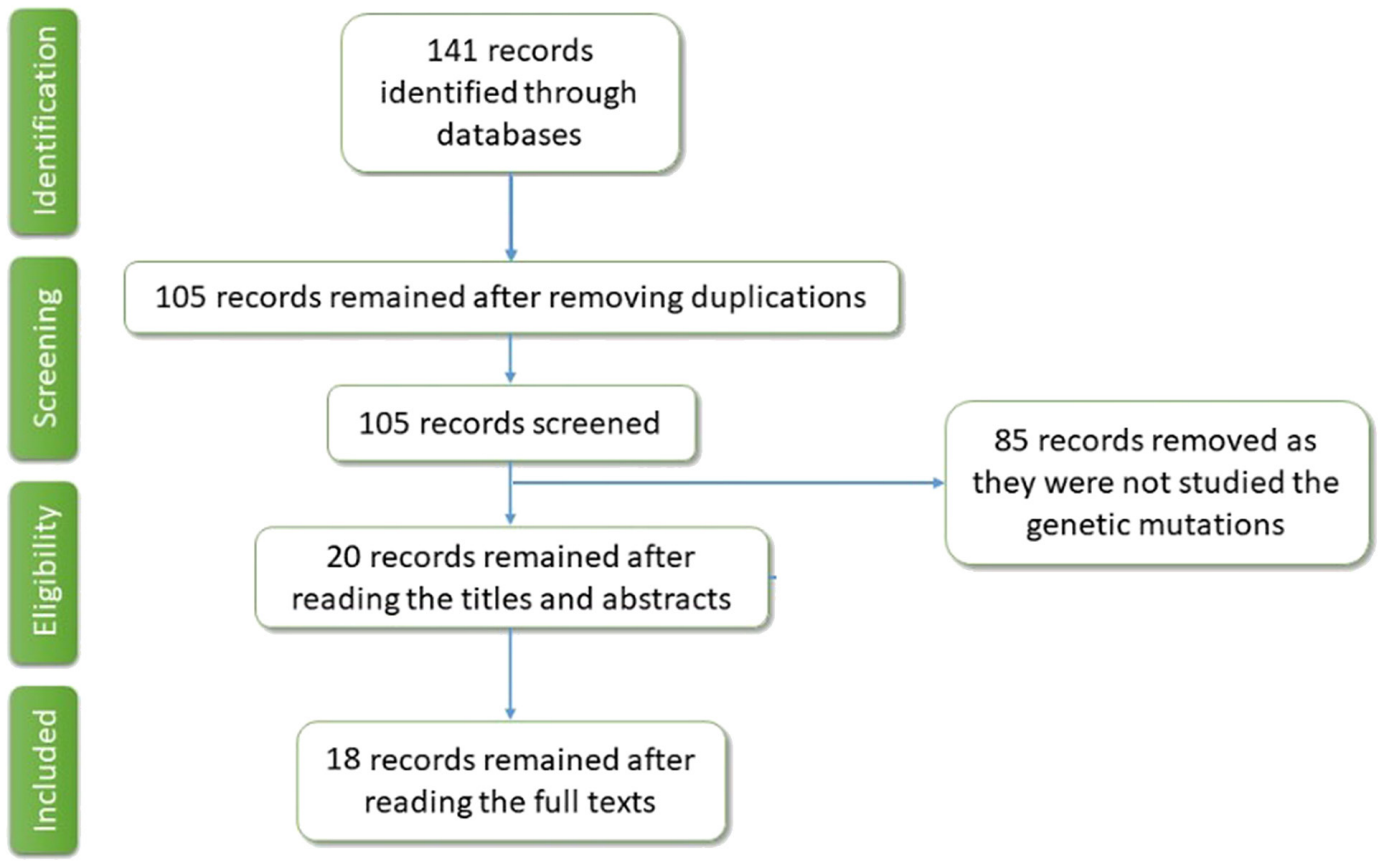
Study selection flow chart.

This literature search for phenylketonuria in Iran yielded totally 105 records without duplicate covering genetic, epidemiology, dietary management, growth and developmental aspects of PKU in Iran. The final number of relevant records related to the genetics of PKU was 18 papers with an appropriate title and abstract (Figure 1).

### 3.1 Patients

From 2003 to 2023, 1497 distinct PAH‐deficient families have been investigated for mutation of *PAH* gene in Iran. Type of marital union has been reported for 1431 families; 968 families (67.65%) had evidence of consanguinity.

### 3.2 Geographic and ethnic distribution of *PAH* mutations

Enrolled studies in this systematic review cover multiple Iranian ethnic groups including Persians, Kurds, Azeris, Lurs, Arabs, Baluchis, Turkmen, Gilak, Tabari, Talesh, Qashqai and tribal groups. The first report of *PAH* mutations in Iranian PKU patients has been performed by Vallian et al.^17^ Overall, 17 different common mutations (frequency ≥1%) and more than 100 less common mutations have been reported in multiple Iranian ethnicities that show broad ranges of the *PAH* gene mutations in different regions of Iran indicating high degree of ethnic heterogeneity for PKU in our population. Table 1 show the common mutations by region and ethnicity found in Iranian patients with PKU and Figure S1. demonstrates common mutations in some of the geographical regions in Iran that characterized by 18 studies. In some regions two studies have been conducted and we pooled these studies for calculating the allele frequency of detected variants. Other studies recruited the patients from different regions of the country without characterising the exact geographical regions.

**TABLE 1:** Common mutations by region and ethnicity found in Iranian patients with PKU.

Population	Ethnicity	*N*	*C*	Most common alleles				Ref.
				Mutation	Frequency (%)	Mutation	Frequency (%)	
Iran^a^	Persian, Turks, Kurd, Lurish, Arabs, Gilaks, Tabari, Balouch	870	604 (69.42%)	c.1066‐11G > A	21	c.168 + 5G > C	8.16	21, 23, 41, 43
				p.Arg261Gln	9	p.Pro281Leu	5.40	
				p.Arg243Ter	4.77	c.969 + 5G > A	3.22	
				c.1089delG	3	p.Arg176Ter	2.36	
Central Iran, especially Esfahan	Not reported	174	107 (61.5%)	c.1066‐11G > A	21.83	p.Arg261Gln	11.5	17, 22
				p.Pro281Leu	15.52	c.115_117delTTC	4.9	
Northeast	Not reported	153	108 (70.58%)	c.1066‐11G > A	12.47	p.Leu48Ser	5.5	20, 27
				p.Val399Val	4.25	p.Arg176Ter	3.92	
				p.Arg243Ter	3.6	p.Ile224Thr	3.6	
				p.Pro281Leu	5.23			
Northwestern Iran	Turks	85	50 (58.82%)	c.1066‐11G > A	26.5	p.Pro281Leu	10	24, 25
				p.Ser67Pro	10	p.Arg261Gln	11.76	
Northern Iran	Mazandarani, Talesh, Turkmens, Gilaks, Turks	91	Unknown	c.1066‐11G > A	20.32	p.Arg261Gln	6.59	34, 35, 36
				p.Arg261Ter	7.14			
Western Iran	Kurd, Lurish	45	35 (77.77%)	c.969 + 5G > A	21.11	c.168 + 5G > C	18.9	38, 42
				c. 843‐5 T > C	8.9	c.1089delG	6.7	
Southwest Iran	Persian, Arab, Lurish, Qashqai	40	31 (77.50%)	c.1066‐11G > A	10	p.Tyr356Ter	8.75	^19^
				p.Ser231Pro	8.75	p.Arg143Ter	6.75	
Qazvin and Zanjan	Not reported	39	20 (51.28%)	p.Arg176Ter	10.25	p.Pro281Leu	10.25	^37^
				p.Arg261Gln	7.69	p.Arg261Terp	5.12	

Abbreviations: *C*, number of consanguineous marriages; *N*, number of patients.

^a^ Four studies have been pooled that recruited patient from different parts of Iran. For central Iran, northeast, northwestern Iran, northern Iran and western Iran. Two conducted studies have been pooled for calculation of the allele frequency.

### 3.3 Spectrum and Frequency of *PAH* mutations in Iran

**FIGURE 2:**
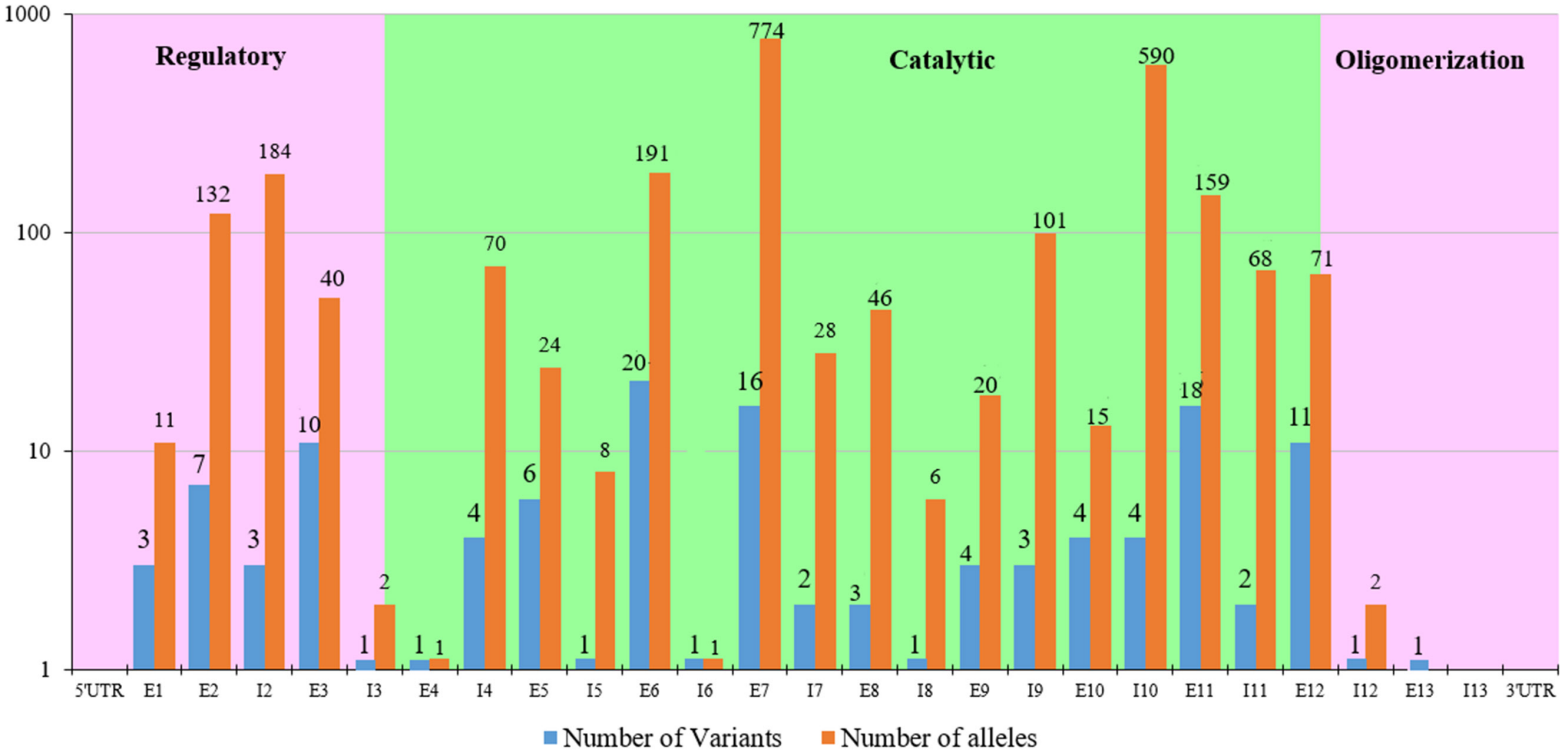
Distribution of *PAH* variants and alleles from 1497 PKU patients from Iran according to gene region and protein domain.

**FIGURE 3:**
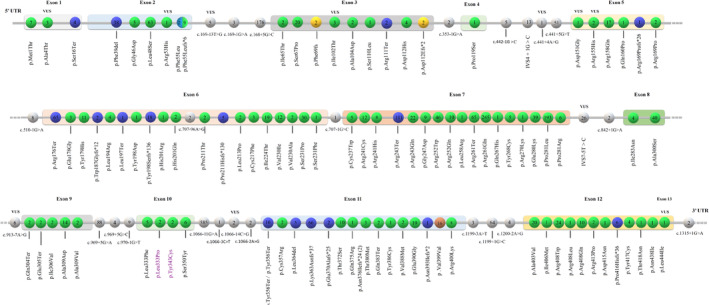
Schematic diagram of *PAH* gene representing exons and introns harbouring mutations identified in Iranian populations. The number in circles above each mutation indicates the number of alleles. The colour of circles shows the type of variant (Blue, deletion; Yellow, insertion; Brown, silent/splicing; Green, missense; Grey, splicing).

By the last update (February, 2023), the mutational spectrum included 129 different mutations (Table S1), which were distributed along almost the entire *PAH* gene sequence and Table 2 provides those mutations that were observed with ≥1% allele frequency. Substitutions were by far the most frequent variant type (85.16%), followed by deletions (11.71%) and insertions (3.125%). Of all variants, 75 were missense (58.13%), followed by 25 splice sites (19.37%) variants and 12 frameshift (9.3%) variants. Nonsense variants (6.97%), large deletions (2.32%), in‐frame variants (1.55%) and synonymous/splicing variants (0.77%) were less frequent (Table 3). Most alleles (79.8%) conveyed mutations that were clustered in exon 7, intron 10, exon 6, intron 2, exon 11 and exon 2 with respective frequencies of 30.42%, 23.19%, 7.50%, 7.23%, 6.25 and 5.18. In the case of *PAH* variant distribution by protein domains, most (77; 60.15%) were located in the central catalytic domain, 22; 17.18% in the N‐terminal regulatory domain, and 6; 4.68% in the C‐terminal oligomerisation domain. The remaining variants (23, 17.96%) were in the intronic regions. No variants were reported in UTR regions; it may be explained by mutation detection rate that were not 100% in studied populations (Figure 2). Of note, this statistical description of *PAH* gene mutations in the Iranian PKU patients is consistent with data in the *PAHvdb* in respect of variant types and the distribution of mutant alleles along the entire *PAH*.^9^ Figure 3 provides the structure of *PAH* gene consisting of 13 exons, with the positions of the variants reported in Iran. The alleles count of each variant is given inside the circles.

**TABLE 2:** Common mutations (AF >1%) found in Iranian patients with PKU.

Mutation		Location	Number of alleles		Mutationtype	Type of PKU
DNA level	Protein level			Frequency %)		
c.1066‐11G > A	–	I10	585	19.54	Splicing	Classic PKU
c.782G > A	p.Arg261Gln	E7	245	8.18	Missense	Classic PKU
c.842C > T	p.Pro281Leu	E7	193	6.45	Missense	Classic PKU
c.168 + 5G > C	–	I2	176	5.88	Splicing	Classic PKU
c.727C > T	p.Arg243Ter	E7	111	3.7	Nonsense	Classic PKU
c.969 + 5G > A	–	I9	88	2.94	Splicing	Classic PKU
c.1089delG	p.K363Nfs*37	E11	66	2.2	Deletion	Classic PKU
c.526C > T	p.Arg176Ter	E6	65	2.17	Nonsense	Classic PKU
c.781C > T	p.Arg261Ter	E7	65	2.17	Nonsense	Classic PKU
c.1199 + 1G > C	‐	I11	64	2.13	Splicing	Classic PKU
c.143 T > C	p.Leu48Ser	E2	63	2.1	Missense	Classic‐mild PKU
c.441 + 5G > T	–	I4	51	1.7	Splicing	Classic PKU
c.754C > T	p.Arg252Trp	E7	46	1.54	Missense	Classic PKU
c.898G > T	p.Ala300Ser	E8	40	1.34	Missense	Mild HPA
c.838G > A	p.Glu280Lys	E7	39	1.3	Missense	Classic PKU
c.115_117delTTC	p.Phe39del	E2	38	1.27	Deletion	Classic PKU
c.691 T > C	p.Ser231Pro	E6	30	1	Missense	Classic PKU
Total			1965	65.61		

**TABLE 3:** Mutation types found in Iranian patients with PKU.

Mutation type	Number	Percent
Missense	75	58.13
Splicing	25	19.37
Deletion	15	11.62
Nonsense	9	6.97
Insertion	4	3.1
Silent/splicing effect	1	0.77
Total	129	100

In terms of frequency of variants, splice site variant in intron 10 (c.1066‐11G > A) was the most prevalent variant throughout Iran possessing 585 alleles with the frequency of about 19.53%. Four other variants with relatively high frequencies were p.Arg261Gln, p.Pro281Leu, c.168 + 5G > C and p.Arg243Ter (8.18%, 6.45%, 5.88% and 3.7%, respectively).

### 3.4 Frequency of c.1066‐11G > A

Pooling 18 studies revealed a wide spectrum of *PAH* variants, with 129 distinct variants present in 2994 alleles. Exon 7, intron 10, exon 6, intron 2, exon 11 and exon 2 contained 79.8% of all the mutant alleles. The remainder of the alleles were distributed along almost the entire *PAH* gene sequence. The pathogenic variant c.1066‐11G > A in intron 10 accounting for 19.53%. of PKU alleles is the most frequent variant among Iranians. Activating an alternative splice site and consequently an in‐frame insertion of nine nucleotides between exon 10 and exon 11 of the processed mRNA and inactivating the catalytic activity of the PAH enzyme,^18^ this variant is widespread throughout Iran and has been reported in several studies carried out in different regions of the country with frequencies varying from 10% to 36%.^19^, ^20^, ^21^, ^22^, ^23^, ^24^, ^25^, ^26^, ^27^

The c.1066‐11G > A as a region‐specific variant has been described as the major PKU‐causing mutation in Middle Eastern and Mediterranean populations such as Turkey,^28^ Italy,^29^ Spain,^30^ Egypt,^31^ Greek^32^ and Israel.^33^ High frequency of c.1066‐11G > A in particular populations can be explained by selective advantage in the heterozygote, recurrent mutation, founder effect and genetic drift; consisting with mentioned mechanisms, population genetic studies have revealed a typical east to the west gradient of this Mediterranean mutation, originating from Western Asia and the Middle East from Armenia via Turkey, Iran and Israel to Spain during the Neolithic period that is line with decreasing frequency rate of c.1066‐11G > A from Turkey to Armenia, Bulgaria, Egypt, Israel, Italy, Tunis and Spain with respective frequencies 32%, 25%, 25%, 17%, 16%, 15%, 12% and 10%.^9^ Beside supporting the fact that Iran have been at the crossroads of human migrations, this Southern Eurasian mutation is an appropriate variant for evolutionary and population genetic studies as well as tracing migration events throughout the world.

### 3.5 Common *PAH* gene variants

The second most common *PAH* pathogenic variant in Iran is p.Arg261Gln (8.18%). The prevalence of this variant was 1.8%–18.25% in Iranian populations. It seems that this mutation is most frequent in Turkish populations. The highest frequency of p.Arg261Gln (18.75%) was reported among Azeri‐Turkish population from West Azerbaijan and this finding can be explained by close familial link between this province and Turks, which is consistent with the historical as well as geographical relations between West Azerbaijan and Turkey.^25^ Of note, p.Arg261Gln with allele frequency of 8.7% is the second most common mutation in Turkey.^28^ This variant has a relatively high allele frequency in some parts of Iran such as Esfahan,^22^ Mazandaran and Golestan,^34^, ^35^ Guilan,^36^ Qazvin and Zanjan,^37^ East Azerbaijan^24^ and less allele frequency in northeast^27^ as well as among Kurds.^38^ The distribution of p.Arg261Gln as a CpG mutation is difficult to explain by ancient or recent migration which has a moderate allele frequency in several South European countries and high frequency in Middle East unconnected by known movement of peoples in the past. Linking to various haplotypes, independent recurrence in two or more founders is possible for p.Arg261Gln.^39^

The third most common variant in Iran is p.Pro281Leu with the frequency of 6.45%. This severe pathogenic variant is a C‐to‐T transition at the second base of codon 281, which is also the last nucleotide in exon 7 of the *PAH* gene and lead to the substitution of Leu (CTT) for Pro (CCT).

The p.Pro281Leu shows regional variations of allele frequencies in Iran that can reflect ethnic and racial diversities in the geographical context of Iran. In line with the mentioned fact, p.Pro281Leu occurs commonly in Isfahan Province—Central Iran,^22^ Iranian Azeri‐Turkish population^24^ both with the frequencies of 19.3% as well as in Qazvin and Zanjan^37^ and North‐east region of Iran^20^, ^27^ with respective frequencies of 10.25% and 7.24%.^37^ However, this variant was absent in the studies performed on the PKU patients from southwest, western and north regions of Iran.^19^, ^38^, ^40^ As the p.Pro281Leu is prevalent among Turks, the virtual absence of this variant in the study performed in West Azerbaijan using RFLP‐PCR for detection of limited number of mutations may reflect the methodological limitation.^25^

The fourth most common variant in Iran with the frequency of 5.88% and possessing 176 allele of 2994 allele is c.168 + 5G > C mutation. It has been reported in nine studies performed in Iran with different frequencies.^21^, ^22^, ^23^, ^24^, ^27^, ^37^, ^38^, ^41^ Surprisingly, this splicing variant along with c.969 + 5G > A are two most prevalent mutations in Kermanshah province located at west of Iran with mainly Kurdish population.^38^ It seems that, the c.969 + 5G > A variant is also common in Hamadan and Lorestan provinces.^42^ Similar to p.Pro281Leu, absence of c.168 + 5G > C in various studies in Iran can contributed to methodological limitation.

The fifth most common variant in Iranian PKU cohort is p.Arg243Ter (3.70%). This nonsense variant is located in exon 7 of *PAH* gene and its association with V245V polymorphism has been reported by several studies.^19^, ^20^, ^21^, ^24^, ^38^, ^43^ A typical genotype (p.Arg243Ter/p.Ser231Pro/p.Ser231Pro) consisting heterozygote variant of p.Arg243Ter and homozygote variant of p.Ser231Pro has been reported in a patient form southwest of Iran.^19^ Taken together, this study corroborates the high frequency of at least five common variants (1310 alleles, 51.5%,) and its geographical predominance in the northwest, north, northeast and central regions of Iran (Figure S1), probably due to the founder effect and/or genetic drift; this has been corroborated by included studies. However, evidences supports founder variants within the *PAH* gene among various ethnic populations^44^ but further research specially migration studies is needed to support this hypothesis.

### 3.6 Less common and private variants

Sixty‐seven variants were found with frequencies of <1%. Twenty‐four variants (18.75%) were observed in only one allele and one family (private variants, Table S2). Representing high variation of mutational spectrum, these variable compound heterozygous variants shows that the number of different variants in Iran like several other populations in the world is usually high, with a few prevalent variants and a large number of less common variants and also private variants. Furthermore, there are substantial differences in the mutational spectrum among Iranian populations indicating cultural, geographical, ethnical and racial diversity in Iran (Table S1).

### 3.7 Mutational hotspots and arginine mutation

There are 1198 and 48 CpG dinucleotides in the genomic sequence of *PAH* and its cDNA, respectively. These CpG dinucleotides are potential sites for recurrent mutation.^45^ Mutability prediction for the cDNA sequence of *PAH* has shown that the majority of the predicted hypermutable regions coincide with the 24 CpG dinucleotide sites.^46^

Among the 129 *PAH* variants in this study, 20 are known to be CpG‐type alleles; another 2 occur at CpG sites but are not C → T or G → A transitions (Table S3). Taken together, CpG‐type alleles accounts for more than 33.56% (854 alleles) of PKU alleles in Iran. Of note, it has been widely assumed that CpG sites could be hypermutable if the cytosine was methylated (mCpG). Methylation‐mediated spontaneous deamination of 5 mC is assumed to be the mechanism, creating hotspots of point mutation (C > T). It is worth mentioning that the actual methylation profile of cytosine for any ‘hypermutable’ *PAH* allele has not yet been examined to the best of our knowledge.^47^

The 13 exons of *PAH* have 24 arginine coding triplets. Thirteen of 24 of arginine triplets contain CpG dinucleotides. In merged studies from Iran, the changes in the arginine coding sequence, accounting 16.40% (21 different variants) of total reported variants. Notably, 10 of 21 mutated arginine coding triplets containing CpG dinucleotides that 9 are CpG‐type alleles and 1 (c.506G > C, R169P) is non‐CpG‐type allele. Fourteen mutated arginine coding triplets p.Arg241Cys, p.Arg241His, p.Arg241Leu, p.Arg243Ter, p.Arg243Gln, p.Arg252Trp, p.Arg252Gln, p.Arg261Ter, p.Arg261Gln, p.Arg270Lys, p.Arg408Trp, p.Arg408Leu, p.Arg408Gln, p.Arg413Pro occur in exons 7 and 12 (66.66%). Moreover, among 1497 Iranian PKU patients, the percentage of the mutations in the arginine residues is 21.24% (636/2994). Of note, the second most common variant (p.Arg261Gln) among Iranian is arginine coding triplet containing CpG dinucleotide (Table S4).

### 3.8 Genotypes of 1157 PKU families

Among the 1497 patients, for 1157 patients (830 homozygotes, and 273 compound heterozygotes, and 54 heterozygotes 73.68%), 281 different genotypes have been reported. For 54 patients the second allele status is unknown demonstrating that most studies had not analysed entire of the *PAH* gene and the mutation detection rate was not 100%. incomplete mutation detection rate can be attributed to methodological approaches. In terms of homozygous genotype frequency, c.1066‐11G > A/c.1066‐11G > A was the most prevalent genotype throughout Iran affecting 214 patients with the frequency of about 18.5%. Four other most common homozygous genotypes were p.Arg261Gln/p.Arg261Gln (83 patients; 7.17%), p.Pro281Leu/p.Pro281Leu (78 patients; 6.74%), c.168 + 5G > C/c.168 + 5G > C (65 patients; 5.61%), and p.Arg243Ter/P. p.Arg243Ter (37 patients; 3.19%), (Table S5). Table 4 shows the 10 common genotypes in Iranian PKU patients. Among the 170 compound heterozygous genotypes, the p.Arg261Gln/c.1066‐11G > A with the frequency of 1.12% (13 patients) was relatively common genotype.

**TABLE 4:** Most common genotypes among Iranian PKU patients.

Genotype	Allele 1	Allele 2	Number of patients	Frequency (%)	Pre‐treatment serum Phe levels (mg/dL)
1	c.1066‐11G > A	c.1066‐11G > A	214	18.5	4.9–80
2	p.Arg261Gln	p.Arg261Gln	83	7.177	3.0–33
3	p.Pro281Leu	p.Pro281Leu	78	6.74	9.0–40
4	c.168 + 5G > C	c.168 + 5G > C	65	5.61	5.8–32
5	p.Arg243Ter	p.Arg243Ter	37	3.19	3.3–46
6	c.969 + 5G > A	c.969 + 5G > A	31	2.67	6.6–54
7	p.Arg176Ter	p.Arg176Ter	24	2.07	13–63.5
8	c.1199 + 1G > C	c.1199 + 1G > C	23	1.98	12–23.5
9	p.Arg261Ter	p.Arg261Ter	23	1.98	9.9–26
10	p.K363Nfs*37	p.K363Nfs*37	23	1.98	3.8–33
11	c.441 + 5G > T	c.441 + 5G > T	16	1.38	10.3–45
12	p.Arg252Trp	p.Arg252Trp	15	1.3	9.3–23
13	p.Glu280Lys	p.Glu280Lys	15	1.3	18–35
14	p.Phe39del	p.Phe39del	15	1.3	14.70–16
15	p.Arg408Trp	p.Arg408Trp	11	0.95	14.5–25
16	p.Ser231Pro	p.Ser231Pro	10	0.86	10.5–23

### 3.9 BH4 response prediction

According to the genotype, BH4 responsiveness can be predicted for PKU patients.^48^ Mutations with residual protein activity of 10% or less were classified as ‘severe’ with negative BH4 responsiveness. Mutations with a residual protein activity of more than 10% were classified as ‘mild’ with a probable responsiveness to BH4.^9^ Patients with two ‘severe’ mutations in the *PAH* gene were considered nonresponders to the BH4 therapy. Patients with at least one mild mutation were potentially sensitive to the therapy. It should be noted that in Iran, it is acceptable to initiate the treatment regardless of patient's genotype. Evaluation of the genotype role in predicting the most probable responsive individuals are limited in Iran. Recently, as a first such evaluation, Khaghani et.al reported 13 different BH4 responsiveness genotypes in PKU patients from northeast of Iran. In this study, it has been demonstrated that at least one non‐null pathogenic variant is most probable to show mild responding phenotype. Therefore, patients with at least one non‐null pathogenic variant are more likely to respond to BH4.^49^

## 4 CONCLUSION

The aim of this study was to elucidate the distribution of causative *PAH* variants in Iran. We provided an estimation of allele frequency for 129 identified variants among Iranian populations. A comprehensive investigation of the mutation spectrum of the *PAH* gene in a given population not only can facilitate genetic counselling for patients and their families but also could be advantageous for establishment of the accurate diagnosis and planning of dietary‐based treatment and other therapeutic strategies as well as guiding the multidisciplinary teams towards a more effective patient follow‐up. As the most diagnostic laboratories in Iran first target some common variants for mutation detection in monogenic disorders and they continue this hierarchical mutation screening strategy for the entire of the gene, the characterisation of common mutations enables creation of diagnostic panels for DNA diagnostics of PKU with lower material and technical costs for diagnostic laboratories that first target some common variants for mutation detection. So, it is recommended that a step‐wise genetic analysis approach be performed with priority sequencing for exon 7, intron 10, exon 6, intron 2, exon 11 and exon 2. In this study, we have tried to describe the molecular basis of PKU and present an update of the mutational landscape of this common metabolic disorder in Iran. The second‐biggest country in the Middle East, Iran, located south of the Caspian Sea and north of the Persian Gulf is a multi‐ethnic country of more than 80 million inhabitants that has a higher incidence of PAH‐deficient PKU than in either Turkey, the United States or Europe.^16^ Consanguineous marriage, as a social and cultural norm in Iran, has led to an increased incidence of PKU. Moreover, inbreeding within the same ethnic and religious group has also contributed to an increased disease incidence. Although an Iranian national program for screening and prevention of PKU has established in recent years,^41^ there is still delayed diagnosis and the quality of nutritional management are additional issues are both consequences of inadequate PKU centres and the long‐distance patients must often travel to obtain medical diagnosis or care. Early diagnosis via effective newborn screening and gain additional information and guidance on the path for follow‐on diagnostic procedures to classify prospectively identified PKU newborns in the Iranian population is an evident need.

In summary, this study was undertaken to produce a detailed picture of the mutational landscape underlying PKU in the Iranian population. Although a relatively high diagnostic rate was achieved by aggregating data, different mutation detection rate in different studies and putative duplications of patients that participate in more than one study may have caused some bias in the frequency of *PAH* variants. Finally, by merging the data of 18 studies done from 2003 to 2023, the cumulative data set yields insights into factors, which have shaped the population of the Iran and migration events between Iran and Middle Eastern and Mediterranean countries, underlining the utility of recessive disease‐associated alleles in the study of human populations.

## AUTHOR CONTRIBUTIONS

**Naser Ajami:** Conceptualization (equal); data curation (equal); formal analysis (equal); methodology (equal); writing – original draft (equal). **Anvar Soleimani:** Formal analysis (supporting); investigation (supporting); methodology (supporting). **Reza Jafarzadeh‐Esfehani:** Methodology (equal); resources (equal). **Mojtaba Hasanpour:** Data curation (equal); formal analysis (equal); writing – review and editing (equal). **Romina Rashid Shomali:** Funding acquisition (equal); resources (equal); validation (equal); writing – review and editing (equal). **Mohammad Reza Abbaszadegan:** Supervision (equal); validation (equal); writing – review and editing (equal).

## CONFLICT OF INTEREST STATEMENT

The authors have no conflict of interest to declare.

## Supporting information


Figure S1

Table S1

Table S2

Table S3

Table S4

Table S5

Data S1

## Data Availability

Data available on request from the authors.
