# Supplementary material for: Mutational landscape of phenylketonuria in Iran
Source: J Cell Mol Med. 2023 Jul 31;27(17):2457–66. doi: 10.1111/jcmm.17865 (PMC10468661; doi:10.1111/jcmm.17865)
Supplement: Supplementary file 1 — Figure S1 [file JCMM-27-2457-s002.docx]

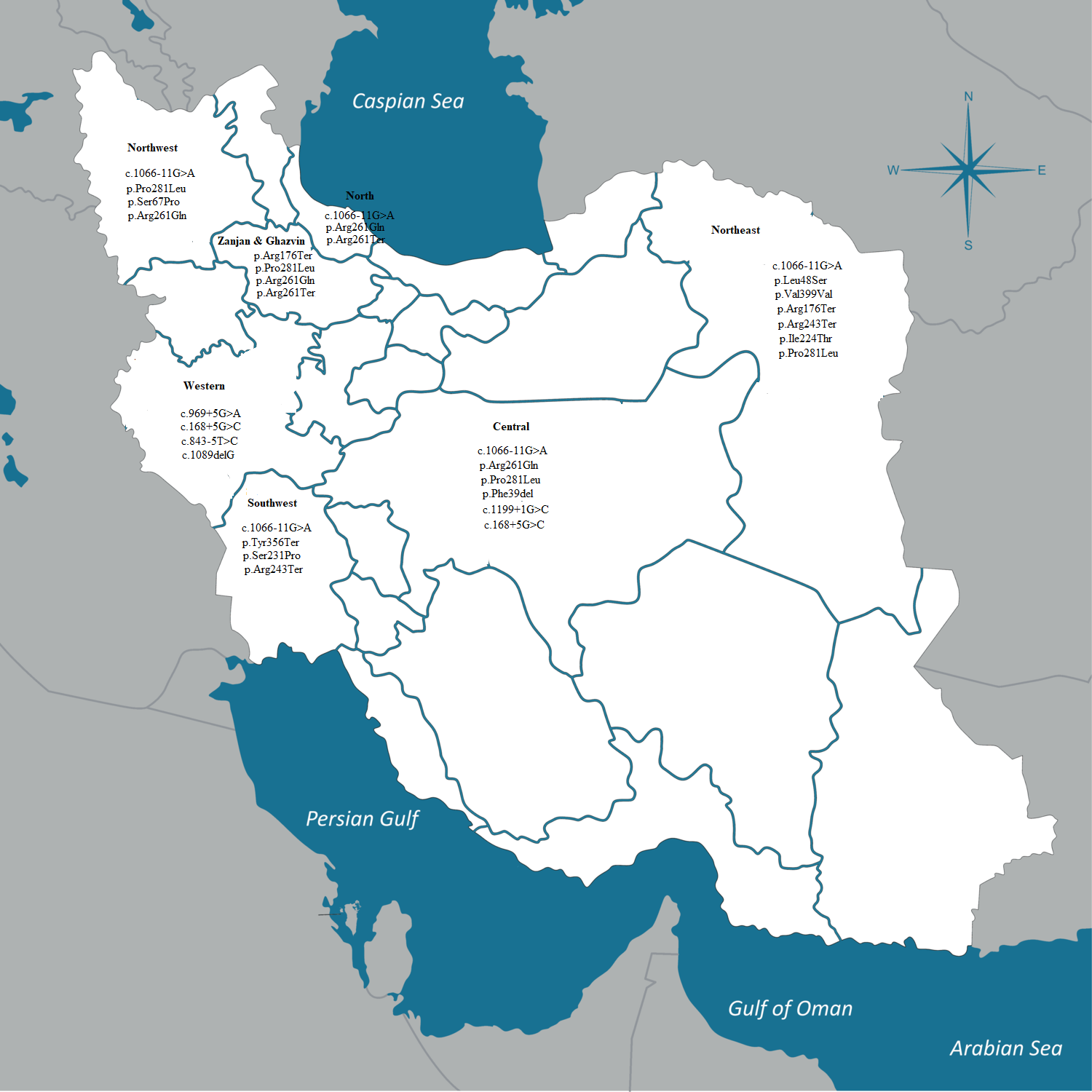


Figure S1 common mutations in some of the geographical regions in Iran that characterized by some studies. In some region two studies have been conducted and we pooled these studies for calculating the allele frequency.
