# Supplementary material for: Mutational landscape of phenylketonuria in Iran
Source: J Cell Mol Med. 2023 Jul 31;27(17):2457–66. doi: 10.1111/jcmm.17865 (PMC10468661; doi:10.1111/jcmm.17865)
Supplement: Supplementary file 3 — Table S2 [file JCMM-27-2457-s003.docx]

Table S2 Private variants found in Iranian patients with PKU

| Ethnicity | Mutation  type | Zygosity | Location | mutation | |  |
| --- | --- | --- | --- | --- | --- | --- |
|  |  |  |  | Protein level | DNA level |  |
| Persian | Deletion | cHet | E1-3 | c.(1-1393_1-405)_(352+33_370)del | |  |
| Kurd | Missense | cHet | E2 | p.F55L | c.165T>G |  |
| Turks | Deletion | Hom | E3 | p.Ex3del4765 | g.21560_26324del476 |  |
| Persian | Missense | Hom | E3 | p.I65T | c.727C>T |  |
| Persian | Missense | cHet | E3 | p.S110L | c.329C>T |  |
| unknown | Insertion | cHet | E3 | p.D112Efs*2 | c.335dup A |  |
| Turks | Splicing | Hom | I3 | - | c.353-1G>A |  |
| unknown | Missense | cHet | E4 | p.P119S | c.355C>T |  |
| unknown | Splicing | cHet | I4 | - | c.441+4A>G |  |
| Gilaki | Missense | cHet | E5 | p.D151G | c.452A>G |  |
| Persian | Missense | Hom | E5 | p.R155H | c.464G>A |  |
| unknown | Missense | cHet | E5 | Q160P | c.479A > C |  |
| Persian | Frame shift | cHet | E5 | p.R169Pfs*26 | c.506delG |  |
| Persian | Frame shift | Hom | E6 | p.W187Gfs*12 | c.558_559delAT |  |
| Unknown | Missense | Hom | E6 | p.L194R | c.581G>T |  |
| Unknown | Deletion | cHet | E6 | p.L197* | c.590_612del23 |  |
| Persian | Missense | cHet | E6 | p.Y198D | c.592T>G |  |
| Tabari | Missense | cHet | E6 | p.H201R | c.602A>G |  |
| Turks | Missense | cHet | E6 | p.P211T | c.631C>A |  |
| Persian | Missense | Hom | E6 | p.L213P | c.638T>C |  |
| Persian | Missense | Hom | E6 | p.V230A | c.689T>C |  |
| Persian | Missense | cHet | E6 | p.S231F | c.692C>T |  |
| Persian | Splicing | cHet | I6 | - | c.707-1G>C |  |
| Tabari | Missense | cHet | E7 | p.L258R | c.773T>G |  |
| Persian | Missense | cHet | E7 | p.Q267H | c.801G>T |  |
| Turks | Missense | CHet | E7 | R270K | c.809G>A |  |
| Unknown | Splicing | Hom | I7 | - | c.842+1GNA |  |
| Unknown | Missense | Hom | E8 | p.I306V | c.916A>G |  |
| Persian | Missense | Hom | E9 | p.A309V | c.926C>T |  |
| Persian | Missense | Hom | E10 | p.Y343C | c.1028A>G |  |
| Turks | Splicing | Hom | I10 | - | c.1066-14C>G |  |
| Persian | Splicing | cHet | I10 | - | c.1066-3C>T |  |
| Persian | Splicing | Hom | I10 | - | c.1066-2A>G |  |
| Persian | Frame shift | Hom | E11 | p.E370Afs*25 | c.1106-1107insAGCT |  |
| Arab | Missense | cHet | E11 | Q375R | c.1124A>G |  |
| Unknown | Missense | Hom | E11 | p.Q383X | c.1147C>T |  |
| Persian | Missense | cHet | E11 | p.Y386C | c.1157A>G |  |
| Persian | Frame shift | cHet | E11 | p.N393Ifs*2 | c.1177-1178InsT |  |
| Persian | Missense | cHet | E12 | p.I406M | c.1218A>G |  |
| Unknown | Missense | cHet | E12 | p.R408L | c.1223G>T |  |
| Persian | Missense | cHet | E12 | p.R413P | c.1238G>C |  |
| E: Exon, I: Intron, cHet: Compound heterozygote, Hom: Homozygote | | | | | | |
