# Supplementary material for: Mutational landscape of phenylketonuria in Iran
Source: J Cell Mol Med. 2023 Jul 31;27(17):2457–66. doi: 10.1111/jcmm.17865 (PMC10468661; doi:10.1111/jcmm.17865)
Supplement: Supplementary file 7 — Data S1 [file JCMM-27-2457-s001.docx]

**Table S1** Mutational spectrum of PAH gene in Iran

**Table S2** Private variants found in Iranian patients with PKU

**Table S3** CpG-type variants, C→T or G→A transition mutations during deamination of the 5’ methylcytosine

**Table S4** Arginine variants

**Table S5** Iranian PKU Genotypes
